# Omega-3 fatty acids for the treatment of depressive disorders in children and adolescents: a meta-analysis of randomized placebo-controlled trials

**DOI:** 10.1186/s13034-019-0296-x

**Published:** 2019-09-14

**Authors:** Li Zhang, Huan Liu, Li Kuang, Huaqing Meng, Xinyu Zhou

**Affiliations:** 1 The First Branch, The First Affiliated Hospital of Chongqing Medical University, Chongqing, China; 2 Department of Psychiatry, The First Affiliated Hospital of Chongqing Medical University, Yixueyuan Road, Yuzhong District, Chongqing, 400016 People’s Republic of China

**Keywords:** Omega-3 fatty acids, Pediatric, Depression, Meta-analysis

## Abstract

**Background:**

To investigate the efficacy and safety of omega-3 fatty acids (O3FA) in treating depressive disorders in children and adolescents.

**Method:**

We conducted a comprehensive search in electronic databases and hand-searched articles included for relevant studies. We included randomized controlled trials which studied on O3FA for treatment of children and adolescents with depression. The standard mean differences (SMDs) and the odds ratios (ORs) with 95% confidence intervals (CIs) were estimated by a random-effects model. The primary outcomes were end-point depressive symptoms scores (efficacy) and all-cause discontinuation (safety). The secondary outcome of response rate was also assessed. Subgroup analyses were performed by age, severity of depression and dosage. Risk of bias assessment was performed based on the Jadad score and the Cochrane Collaboration’s risk-of-bias method.

**Results:**

A total of four studies with 153 participants were included. In terms of efficacy, there was no significant difference of end-point depressive symptoms scores between O3FA and placebo (SMD = − 0.12, 95% CI − 0.53 to 0.30, P = 0.58; *I*^2^= 30%). In terms of safety, the all-cause discontinuation showed no statistical significance between O3FA and placebo (OR = 1.3, 95% CI 0.58 to 2.93, P = 0.53; *I*^2^= 0%). The response rate of O3FA was also not significant better than that of placebo (OR = 1.57, 95% CI 0.26 to 9.39, P = 0.62; *I*^2^= 71%). Besides, there were also no significant differences in those subgroup analyses outcomes. The risk of bias of included trials were not high.

**Conclusions:**

Only considering the limited evidence of O3FA in the acute treatment of major depressive disorder, it did not seem to offer a clear advantage for children and adolescents.

## Background

Depression is a common and serious mental disorder. As reported, there are more than 350 million depressed people all over the world [1]. As to pediatric depression, the prevalence is also high, with approximately 2.8% of children and 5.6% of adolescents worldwide [2]. A 70% chance of pediatric depression will relapse in 5 years, and half of young people would experience a recurrence at least once during their adult life [3]. Pediatric depression is always under-diagnosed, because they may have only atypical depressive manifestations, such as irritability, mood fluctuating, and school refusal [4, 5]. Depression does great harm to young people’s social ability, and it is a major risk factor for suicide in children and adolescents [1, 6]. There are mainly two therapies: psychotherapy and pharmacotherapy. Although psychotherapy is recommended as the first-line treatment for depression in children and adolescent [6], the effect is always mild [4]. Antidepressants are widely used in clinic and for moderate to severe pediatric depression, antidepressants and psychotherapy may be started concurrently [6]. However, in 2016, a network meta-analysis including 34 randomized placebo-controlled trials (RCTS) concluded that most antidepressant drugs did not seem to offer a clear benefit to pediatric depression [7]. And as early as in 2004 the US Food and Drug Administration (FDA) alerted clinicians to the increased risk of suicidality (suicidal thinking and behavior) in children and adolescents associated with antidepressants use [8].

Omega-3 fatty acids (O3FA), a kind of nutrients, is composed of eicosapentaenoic acid (EPA) and docosahexaenoic acid (DHA), which cannot be synthesized efficiently by human body, so dietary intake is the main source, such as fish oil, seafood, flaxseed oil and perilla oil [9]. Recently, researches found that O3FA supplementation might be effective for several neuropsychiatric disorders, such as attention deficit hyperactivity disorder (ADHD) and autism spectrum disorder (ASD) [10–12]. It was also reported that higher fish consumption was related to a reduced depression risk [13, 14] and O3FA was an effective adjunctive treatment for adult depression [15, 16]. Several meta-analyses and reviews also showed that supplementation of O3FAs could relieve symptoms of depression for adult age groups [17–19], but no such evidence especially studied in depressed children and adolescents. Therefore, we conducted this meta-analysis to pool present evidences on efficacy and safety of O3FA compared to placebo in the treatment of children and adolescents with depressive disorders.

## Method

### Literature search

We conducted a comprehensive search in the following electronic databases, including PubMed, Embase, Cochrane Library, Web of Science, and PsycINFO citations, as well as some international trials registers, including WHO’s trials portal, US ClinicalTrials.gov, EU Clinical Trials Register and Australian New Zealand Clinical Trials Registry, up to July 2019. The following search terms were used: (‘omega-3’ or ‘n − 3’ or ‘polyunsaturated fatty acid*’ or ‘unsaturated fatty acid*’ or ‘PUFA’ or ‘eicosapentaenoic acid’ or ‘docosahexaenoic acid’ or ‘EPA’ or ‘DHA’) and (‘child*’ or ‘adolesc*’ or ‘pediatri*’) and (‘depress*’ or ‘dysthymi*’ or ‘affective disorder*’ or ‘mood disorder*’). Relevant articles were also hand-searched for eligible reports. No limitations were applied in the search.

### Selection criteria

We included: (1) RCTs with both parallel arms and cross-over design (for cross-over trials, we only used data from the pre-crossover phase); (2) children (aged 6–12) and/or adolescents (aged 13–18) with depressive disorders; (3) the intervention group could be O3FA treatment, or any component of it (EPA or DHA). No combined treatments like antidepressants or psychotherapy; (4) the comparison group should be placebo treatment; (5) efficacy outcome was assessed by depression scales. The most common questionnaire or instrument used in the youth are The Children’s Depression Rating Scale (CDRS), revised CDRS (CDRS-R), Beck Depression Inventory (BDI) and Children’s Depression Inventory (CDI). We used the end-point score of depressive scale in each group as our primary efficacy outcome. The secondary efficacy outcome was the response rate to omega-3 treatment. The response rate was defined as ≥ 50% change from baseline on depression score or a score of ≤ 28 at the end-point of a trial on the CDRS-R [20]. We also investigated all-cause discontinuation as safety outcome. We excluded: (1) trials without random design or with just quasi-random design; (2) data of outcomes couldn’t be acquired; (3) studies with duplicated data. Two authors (ZL and ZXY) reviewed all the screened trials independently according to the above inclusion and exclusion criteria with strong interrater agreement (***κ ***= 0.90).

### Data collection and risk of bias assessment

The following data were collected: publication information (the first author, publication year, study country), study and patients characteristics (study design type, sample size, age group, diagnostic criteria, severity of depression, rating scales, daily dosage and duration of O3FA, ratio or dosage of EPA and DHA), outcome data (baseline data, post-treatment data, drop-out rate, adverse events).

Risk of bias of the selected studies was assessed by the modified Jadad score [21] and the Cochrane Collaboration’s risk-of-bias method [22] simultaneously. According to the modified Jadad score, we appraised risk of bias from four domains, including generation of allocation sequence, allocation concealment, investigator blindness, and description of withdrawals and dropouts. The specific scoring method was shown in Additional file 1: Figure S1.

All of the above data extraction and risk of bias assessment were finished by the two reviewers (ZL and ZXY) independently. When meeting missing data or information, one author would e-mail the authors for further acquisition. Disagreements were resolved by discussion.

### Statistical analysis

RevMan 5.3 version software and Stata 13.0 were used to perform all the analyses in the meta-analysis. We adopted standard mean differences (SMDs) with 95% confidence intervals (CIs) to estimate effect size of continuous variables and the odds ratios (ORs) with 95% CIs to estimate effect size of dichotomous variables. For continuous variables, difference of the end-point data with standard deviation (SD) between O3FA and placebo was the effect value [23]. A random-effects model was chosen to calculate the effect sizes for expected heterogeneity. If SD was unavailable in a article and could not contact the authors, we would be calculate it from reported P values, t values, CIs or standard errors (SEs) in the article [24]. The heterogeneity was calculated by the test of inconsistency (*I*^2^) [25]. To investigate the possible sources of heterogeneity, we conducted subgroup analyses. The publication bias was evaluated by Egger tests when there were more than ten trials [26]. A two-sided P value of less than 0.05 was considered statistically significant.

## Results

### Selection of studies

**Fig. 1:**
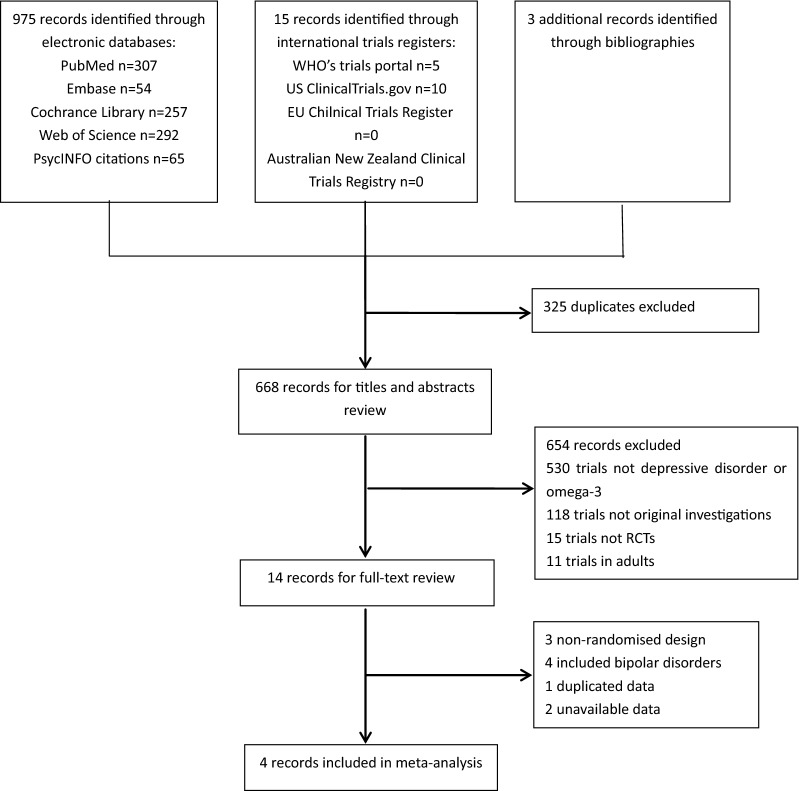
Flow diagram indicating the process of selecting eligible studies

With the keywords above, a total of 993 records was yielded preliminarily, of which 990 records were from electronic databases and three records were from hand-search. After removing the 325 duplicates, 668 records were reviewed based on titles and abstracts. And then, 14 potentially eligible records were screened out for full-text review. With careful review and strict criteria, we finally included four RCT trials in this meta-analysis [27–30]. The flow diagram was shown in Fig. 1. The 14 excluded records were shown in Additional file 2: Table S1.

### Description of the included studies

Most of the included studies were published in recent 2 years except the one by Nemet [29]. Of the four included studies, two were from America [27, 28], whereas one from Europe [30] and the remaining one from Asia [29]. Two studies were conducted in children [27, 29], while the other two were performed in adolescents [28, 30]. However, mean sample size was 38 participants, in which only one study by Gabbay [28] recruited more than 50 participants. Most participant experienced moderate to severe depressive symptoms at baseline on the depression rating scales. In the intervention groups, all the participants received O3FA with a fixed ratio of EPA to DHA and all the ratios of EPA to DHA were higher than 1:1, but there was still a significant difference in the daily intake between studies (400 mg/day to 2289 mg/day). None of the studies provided a single ingredient oil. The whole treatment duration was relatively long with a mean duration of 12.5 weeks. Three studies chosen CSDR or CSDR-R [27–29] and one study applied CDI [30] to assess improvement in depressive symptoms. The characteristics of the included studies were shown in Table 1.

**Table 1:** Characteristics of the four included studies

Study	Country	N^a^	Male (%)	Age group (mean)	Disease	Severity	Diagnostic criteria	O3FAs daily dosage (g/d)	EPA daily dosage (g/d)	Ratio of EPA:DHA	Duration (weeks)	Rating scale	End-point scores		Jadad score^e^
													O3FAs arm	Placebo arm	
Fristad 2016 [27]	USA	36 (18/18)	61.1	7–14 y (11.7)	Depressive disorder	Mild to moderate	DSM-IV-TR	1.9	1.4	7:1	12	CDRS-R	31.0 ± 9.0	31.0 ± 11.0	5
Gabbay 2018 [28]	USA	51 (24/27)	41.7	12–19 y (16.1)	MDD	Moderate to severe	DSM-IV-TR	3.4^b^	2.3^c^	2:1	10	CDRS-R	36.5 ± 10.0	35.2 ± 10.6	4
Nemet 2006 [29]	Israel	28 (13/15)	75.0	6–12 y (10.1)	MDD	Severe	K-SADS	1	0.4 or 0.38^d^	2:1	16	CDRS	32.0 ± 20.5	53.6 ± 20.5	2
Trebaticka 2017 [30]	Slovakia	38 (19/19)	21.1	11–17 y (15.6)	Depressive disorder or mixed anxiety and depressive disorder	Moderate to severe	ICD-10	2.4	1	1.33:1	12	CDI	20.5 ± 11.8	20.3 ± 10.5	4

*CDI* Children’s Depression Inventory, *CDRS-R* Children’s Depression Rating Scale-Revised, *DHA* docosahexaenoic acid, *DSM-IV-TR* Diagnostic and Statistical Manual of Mental Disorders, text revision of the 4th version, *EPA* eicosapentaenoic acid, *ICD* International Classification of Diseases, *K-SADS* Schedule for Affective Disorders and Schizophrenia, the kiddie version, *MDD* major depressive disorder, *NR* not reported, *y* years

^a^The number of patients who were assigned randomly

^b^Mean end daily dosage of O3FA

^c^Mean end daily dosage of EPA

^d^There are two different doses of capsules, one of which was 0.5 g containing 0.19 g EPA and the other one was 1 g containing 0.4 g EPA

^e^The Jadad total score 1–3 indicates low quality, 4–7 indicates high quality

### Risk of bias in the included studies

Generally, the quality of the included studies were not high. In the study by Nemets [29], the capsule used in the O3FA group was different from the one used in the placebo group in tone of internal color. This could result in failure in blinding of intervention. We found the number of response in the placebo group was 0 in that study, which might be biased caused by failure in blinding of intervention. The result of the modified Jadad scores was shown in Table 1. The study quality assessed by the Cochrane Collaboration’s risk-of-bias method was shown in Additional file 3: Figure S2.

### Results for outcomes

**Fig. 2:**
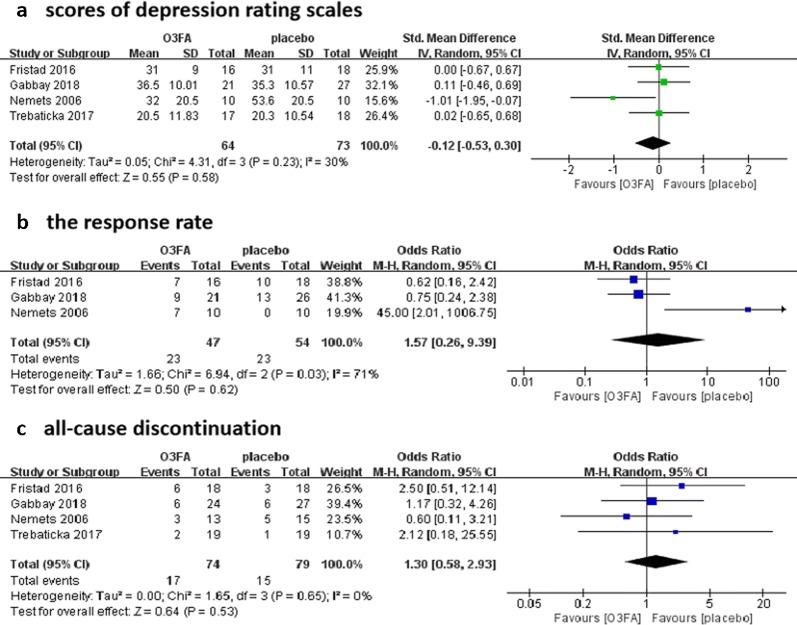
Forest plots for the outcomes compared O3FA with placebo. **a** Scores of depression rating scales; **b** the response rate; **c** all-cause discontinuation

A total of four studies with 153 participants evaluated the efficacy and safety of O3FA for depressive disorders in children and adolescents [27–30]. In terms of efficacy outcomes, the summary effect size of end-point depression scale scores, indicated that O3FA was not better than placebo in treating children and adolescents with depressive disorders, with a SMD of − 0.12 (95% CI − 0.53 to 0.30, P = 0.58; *I*^2^= 30%, P = 0.23; Fig. 2a). The other efficacy outcome we were concerned about, the response rate, was also reported in three studies [27–29]. The response rate of O3FA group was still not superior compared to that of placebo group with a OR of 1.57 (95% CI 0.26 to 9.39, P = 0.62; *I*^2^= 71%, P = 0.03; Fig. 2b). In terms of safety outcome, the OR for the all-cause discontinuation was 1.3 (95% CI 0.58 to 2.93, P = 0.53; *I*^2^= 0%, P = 0.65; Fig. 2c), which meant no statistical significance between the O3FA group and placebo group.

Subgroup analyses were also performed in the primary efficacy outcome, stratified by mean age group (≤ 12 years and > 12 years), severity of depression (mild and moderate to severe), and daily dosage of EPA (≤ 1 g/day and > 1 g/day). No significance were found in those subgroups. Results of subgroup analyses were presented in Table 2. However, as to small number of included studies, we couldn’t conducted sensitivity analysis or evaluated the publication bias.

**Table 2:** Subgroup analyses of O3FA for the treatment of depressive disorders in children and adolescents

Subgroups	Overall effect		Subgroup difference	
	SMD (95% CI)	*P*	*I*^2^ (%)	*P*
Mean age (years)^a^				
≤ 12 [27, 29]	− 0.45 (− 1.43, 0.53)	0.37	0	0.34
> 12 [28, 30]	0.07 (− 0.36, 0.51)	0.74		
Severity of depression				
Mild [27]	− 0.00 (− 0.96, 0.67)	1.00	0	0.67
Moderate to severe [28–30]	− 0.19 (− 0.79, 0.40)	0.52		
Daily dosage of EPA (g/day)				
≤ 1 [29, 30]	− 0.44 (− 1.44, 0.56)	0.39	0	0.36
> 1 [27, 28]	0.07 (− 0.37, 0.50)	0.76		

*EPA* eicosapentaenoic acid

^a^Children were aged between 6 and 12 years and adolescents were aged between 13 and 18 years

## Discussion

To our best knowledge, this was the first meta-analysis focused on the efficacy and safety of O3FA in children and adolescents with depressive disorders. Through a comprehensive search, we finally enrolled four eligible RCTs with 153 participants. According to the results, O3FA had no positive effects in treating depression in children and adolescents with no statistical significance. These results were in contrast to several previous meta-analyses specifically in adults [18, 19, 31, 32]. But these meta-analyses in adults presented great heterogeneity between studies ranging from 64 to 84.1%, which was mainly from different populations, diagnostic criteria and interventions. Of the four included studies in this meta-analysis, only one study by Nemets [29] had reported a beneficial efficacy of O3FA in the treatment of depression in children and adolescents. In that study, the response rate in the placebo group was 0, which was rare in clinical trials in depression among children and adolescents and might have magnified the efficacy of O3FA for children and adolescents. Data from that trial could hardly be generalized. What’s more, diagnostic criteria, severity of depression, daily dosage of EPA and DHA were all heterogenous and trials were small scaled, so these results should be interpreted with cautions. Eicosapentaenoic acid (EPA) was reported to be responsible for the beneficial effects of O3FA in treating depression in adult [33] and was recommended a higher ratio than 1:1 when used combined EPA + DHA [34, 35]. In this review, we did not find greater benefits in studies with higher dose supplementation of EPA in young people.

O3FA is associated with brain development and function [36], which involve in maintaining membrane fluidity, influencing neurotransmission, decreasing levels of inflammatory mediators and affecting cognition function [17, 37, 38]. The study by Grayson et al. had shown that DHA is crucial for visual pathway connectivity and large-scale brain organization [39]. Thus, O3FA was widely investigated in neuropsychiatric disorders. Children and adolescents with ADHD had a deficiency in O3FA levels [40] and supplementation of O3FA could relieve clinical symptoms of ADHD in these young people [10, 12]. Kean et al. [41] conducted a randomised, double-blind, placebo-controlled study which investigated the effects of marine oil extract on symptoms of ADHA in children. The results indicated that marine oil extract may be a preferable alternative treatments for children with ADHD who have just mild or subclinical hyperactivity, inattention and impulsivity. Recent two meta-analyses also presented modest effects of O3FA in the reducing symptoms of ADHD children [42, 43]. Amminger et al. [11] found that O3FA could reduce hyperactivity and stereotypy symptoms in children with ASD. However, a review by James et al. [44] had not find any improvements of symptoms after supplementation of O3FA in people with ASD. The study by Woo et al. [45] also found that supplements of O3FA were acceptable in the pediatric eating disorders population.

Psychotherapy, mainly referring to cognitive behavioural therapy (CBT) and interpersonal psychotherapy (IPT), is still recommended as the first-line treatment for children and adolescents depression, unless the symptoms are severe [46–48]. But for the management of an uncomplicated or brief depression, mild psychosocial impairment, to begin treatment with education, support, and case management appears to be equally efficacious to psychotherapy [48, 49]. With regards to antidepressants, fluoxetine is the first-line medication for depression in children and adolescents [7, 47, 49]. However, use of antidepressants is not recommended in mild depressed youth considering serious adverse effects of drugs, and antidepressants are thought appropriate only after an unsuccessful 3-month specific psychological therapy in moderate to severe depressed adolescents [47, 50]. For a child with moderate to severe depression and unresponsive to a 3-month specific psychological therapy, antidepressants should still be prescribed with cautions [47].

O3FA has an excellent safety profile as dietary nutrient. Only one of the 153 participants stated more frequent defecation after taking O3FA [30]. No other adverse events, even any mild discomforts, were reported in the included studies in this review. More than that, no published literature had reported any side effects of O3FA so far. As no participant was discontinued for adverse events, the outcome of discontinuation for adverse events was not assessed. Meanwhile, the OR for all-cause discontinuation indicated no difference between O3FA and placebo.

This review has several limitations. Firstly, number of studies on children and adolescents with depressive disorders was small. Only four studies met our inclusion criteria. And in the only four eligible studies, the sample sizes were really small with the biggest enrollment of 51 participants. This downgraded the strength of evidence directly. Secondly, diagnostic criteria, severity of depression, daily dosage of EPA and DHA were heterogenous in those included studies. However, due to small number of the included studies, the value of *I*^2^ may have limited statistical power in finding heterogeneity. Thirdly, as polyunsaturated fatty acids are common nutrients in our diets, and baseline dietary intake varies in different population [51]. However, none of the included studies had taken this into consideration in the study design.

## Conclusions

The evidence available indicated no efficacy of O3FA for the treatment of children and adolescents. However, for small number of trials and sample sizes, the strength of evidence was weak. Nevertheless, O3FA were safe without adverse events occurring.

## Supplementary information

**Additional file 1: Figure S1.** The modified Jadad score.

**Additional file 2: Table S1.** Reasons for excluding the 10 studies.

**Additional file 3: Figure S2.** Risk of bias assessed by the Cochrane Collaboration’s risk-of-bias method.

## Data Availability

Not applicable.

## Abbreviations

BDI: Beck Depression Inventory
CDI: Children’s Depression Inventory
CDRS: Children’s Depression Rating Scale
CI: confidence interval
DHA: docosahexaenoic acid
EPA: eicosapentaenoic acid
FDA: Food and Drug Administration
O3FA: omega-3 fatty acids
OR: odds ratio
RCT: randomized placebo-controlled trial
SE: standard error
SMD: standard mean difference
