# Supplementary material for: Omega-3 fatty acids for the treatment of depressive disorders in children and adolescents: a meta-analysis of randomized placebo-controlled trials
Source: Child Adolesc Psychiatry Ment Health. 2019 Sep 14;13:36. doi: 10.1186/s13034-019-0296-x (PMC6744624; doi:10.1186/s13034-019-0296-x)
Supplement: Supplementary file 1 — Additional file 1: Figure S1. The modified Jadad score. [file 13034_2019_296_MOESM1_ESM.docx]

**The modified Jadad score**

|  | **Scoring method** |
| --- | --- |
| Generation of allocation sequence | 2 computer-generated random numbers  1 not described  0 inadequate |
| Allocation concealment | 2 central randomization, sealed envelopes or similar  1 not described  0 inadequate or no concealment |
| Investigator blindness | 2 identical placebo tablets or similar  1 not described  0 inadequate or no double-blinding |
| Description of withdrawals and drop-outs | 1 numbers and reasons are described  0 numbers and reasons are not described |

**Total score 1-3 low quality, 4-7 high quality.**
