# Supplementary material for: Omega-3 fatty acids for the treatment of depressive disorders in children and adolescents: a meta-analysis of randomized placebo-controlled trials
Source: Child Adolesc Psychiatry Ment Health. 2019 Sep 14;13:36. doi: 10.1186/s13034-019-0296-x (PMC6744624; doi:10.1186/s13034-019-0296-x)
Supplement: Supplementary file 2 — Additional file 2: Table S1. Reasons for excluding the 10 studies. [file 13034_2019_296_MOESM2_ESM.docx]

| **Table S1 Reasons for excluding the 10 studies.** | |
| --- | --- |
| **Reasons** | **Excluded studies** |
| **Open-label trials(n=3)** | **McNamara RK, et al.** Effects of fish oil supplementation on prefrontal metabolite concentrations in adolescents with major depressive disorder: a preliminary 1H MRS study. Nutr Neurosci. 2016;19:145-55.  **McNamara RK, et al.** Detection and Treatment of Long-Chain Omega-3 Fatty Acid Deficiency in Adolescents with SSRI-Resistant Major Depressive Disorder. PharmaNutrition. 2014;2:38-46.  **Clayton EH, et al.** Reduced mania and depression in juvenile bipolar disorder associated with long-chain omega-3 polyunsaturated fatty acid supplementation. Eur J Clin Nutr. 2009;63:1037-40. |
| **Bipolar disorders(n=4)** | **Wozniak J, et al.** A randomized clinical trial of high eicosapentaenoic acid omega-3 fatty acids and inositol as monotherapy and in combination in the treatment of pediatric bipolar spectrum disorders: a pilot study. J Clin Psychiatry. 2015;76(11):1548-55.  **Arnold LE, et al.** Impact of individual-family psychoeducational psychotherapy (IF-PEP) and omega3 fatty acids on children with bipolar disorder-not otherwise specified or cyclothymic disorder. 2015;17:62.  **Fistad MA, et al.** A randomized controlled trial of individual familypPsychoeducational psychotherapy and omega-3 fatty acids in youth with subsyndromal bipolar disorder. J Child Adolesc Psychopharmacol. 2015;25:764-74.  **Gracious BL, et al.** Randomized, placebo-controlled trial of flax oil in pediatric bipolar disorder. Bipolar Disord. 2010;12:142-54. |
| **Duplicated data(n=1)** | **Stadterman J, et al.** Randomized control trial of omega-3 fatty acids in adolescents with major depressive disorder.J Am Acad Child Adolesc Psychiatry. 2016;55(10 Supplement 1):S168‐9. |
| **Unavailable data(n=2)** | **DelBello M, et al.** A double-blind placebo-controlled study of long-chain omega-3 fatty acid supplementation for depression in youth at ultra-high risk for bipolar disorder. Neuropsychopharmacology. 2013;38:S374‐5.  **McNamara RK, et al.** Effects of long-chain omega-3 fatty acid monotherapy on cortical biochemistry in depressed bipolar offspring: a double-blind placebo-controlled 1H MRS study. Biol Psychiatry. 2014;75:121S. |
