# Supplementary material for: Omega-3 fatty acids for the treatment of depressive disorders in children and adolescents: a meta-analysis of randomized placebo-controlled trials
Source: Child Adolesc Psychiatry Ment Health. 2019 Sep 14;13:36. doi: 10.1186/s13034-019-0296-x (PMC6744624; doi:10.1186/s13034-019-0296-x)
Supplement: Supplementary file 3 — Additional file 3: Figure S2. Risk of bias assessed by the Cochrane Collaboration’s risk-of-bias method. [file 13034_2019_296_MOESM3_ESM.docx]

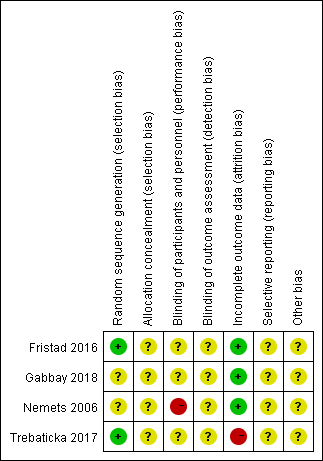


**Fig. S2** **Risk of bias assessed by the Cochrane Collaboration’s risk-of-bias method.**

According to the Cochrane’s recommendations, we appraised risk of bias from six domains, including random sequence generation, allocation concealment, blinding of participants and personnel, binding of outcome assessment, incomplete outcome data, and selective reporting and other bias. Each domain was rated as ‘high bias’, ‘low bias’ or ‘unclear’. In the risk of bias summary figure, we used ‘+’, ‘-’ and ‘?’, to represent ‘high bias’, ‘low bias’ and ‘unclear’, respectively.

In the study by Nemets, the capsule used in the O3FA group was different from the one used in the placebo group in tone of internal color, thus, rating as ‘high bias’ in performance bias domain. In the study by Trebatická, the attribution bias was high because they hadn’t use intention-to-treat analysis.
